# Deeply Cyclable and Ultrahigh‐Rate Lithium Metal Anodes Enabled by Coaxial Nanochamber Heterojunction on Carbon Nanofibers

**DOI:** 10.1002/advs.202101940

**Published:** 2021-10-23

**Authors:** TrungHieu Le, Ciqing Yang, Wei Lv, Qinghua Liang, Xiehe Huang, Feiyu Kang, Ying Yang

**Affiliations:** ^1^ State Key Laboratory of Control and Simulation of Power System and Generation Equipments Tsinghua University Beijing 100084 China; ^2^ Shenzhen Key Laboratory for Graphene‐Based Materials Engineering Laboratory for Functionalized Carbon Materials Tsinghua Shenzhen International Graduate School Tsinghua University Shenzhen 518055 China; ^3^ Department of Chemical Engineering The University of Melbourne Parkville VIC 3010 Australia; ^4^ Laboratory of Advanced Materials Department of Materials Science and Engineering Tsinghua University Beijing 100084 China

**Keywords:** heterojunctions, high lithium utilization, lithium metal anodes, nanochamber structures, ultrahigh current densities

## Abstract

Lithium metal anodes (LMAs) are the most promising candidates for high‐energy‐density batteries due to the high theoretical specific capacity and lowest potential. However, the practical application of LMAs is hampered by the short lifespan and unsatisfactory lithium utilization (<50%). An oxide–oxide heterojunction enhanced with nanochamber structure design is proposed to improve lithium utilization and cycling performance of LMA under ultrahigh rates. Typically, a MnO_2_–ZnO heterojunction provides high binding energy for strong absorption of Li‐ions and intimately bonded interfaces for fast transfer of electrons. Under the guidance of the smooth Li‐ion migration and rapid electron flow, the Li metal can be restricted as thin layers within submicro scale in nanochambers with constrain boundary and stress dissipation, inhibiting the local agglomeration and blocking. Thus, the lithiophilic active sites can be effectively exposed to the Li‐ions within submicro scale, improving the reversible conversion for high lithium utilization during long‐term cycling. As such, the Li@MnZnO/CNF electrode achieves a high lithium utilization of 70% at a record‐high current density of 50 mA cm^−2^ with areal capacity of 10 mAh cm^−2^. This work offers an avenue to improve lithium utilization for long‐lifespan LMAs working under high current densities and capacities.

## Graphical abstract

An oxide–oxide heterojunction with nanochamber structure is proposed to improve lithium utilization of lithium metal anodes. Reversible plating/stripping process and renewable active sites can be guided by fast electron/ion transport paths in the nanochambers that serve as Li‐ion traps and divided buffering space, contributing to high lithium utilization of 70% at a record‐high current density of 50 mA cm^−2^.

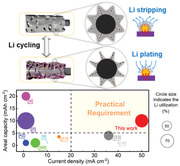

## 1 Introduction

Inrecent years, lithium metal anodes (LMAs) have been widely explored as the most proposing candidates for the next generation of high‐energy‐density batteries due to their high theoretical specific capacity (3860 mAh g^−1^), the lowest electrochemical potential (−3.04 V vs the standard hydrogen electrode), and low mass density (0.59 g cm^−3^).^[^ ^1^, ^2^, ^3^ ^]^ However, the practical applications of LMAs are seriously hindered by low lithium utilization and short lifespan.^[^ ^4^, ^5^, ^6^, ^7^ ^]^ Since the lithium utilization determines the maximum reversible capacity with a limited Li loading, it is highly desired to develop long‐cycling stable and deeply cyclable LMAs (lithium utilization > 50%).^[^ ^5^, ^6^ ^]^ Until now, much achievement has been made to stabilize the LMAs by controlling the Li dendrite growth and buffering volume changes during cycling, but achieving the high lithium utilization under high current densities (>20 mA cm^−2^) or high areal capacity (>5 mAh cm^−2^) is still challenging, which is essential for practical application toward fast charging/discharging rate over 5 C and the energy density goal of 500 Wh kg^−1^ per cell.^[^ ^5^, ^6^, ^7^, ^8^, ^9^, ^10^, ^11^, ^12^ ^]^

Among all the strategies to enhance lithium utilization at high current densities, hosting Li metal in a 3D framework with fast electron transfer is one of the most effective methods. As such, 3D conductive frameworks with various lithiophilic materials such as Au,^[^ ^13^, ^14^, ^15^ ^]^ Ag,^[^ ^10^, ^16^, ^17^ ^]^ ZnO,^[^ ^18^, ^19^, ^20^, ^21^, ^22^ ^]^ and CuO^[^ ^23^, ^24^, ^25^ ^]^ have been developed as LMA hosts. However, an excess of Li will block the pores of the host at a high areal capacity owing to the small ratio of lithiophilic surface area and Li cycling capacity, resulting in the critical reduced nucleation sites with a high lithium utilization larger than 50%. Furthermore, an insufficient exposure of the lithiophilic sites will accelerate Li dendrite growth and dead Li formation without electron contract. Thus, the reversible capacity will decrease rapidly because of the constant Li consumption during the cycling process.

**Figure 1:**
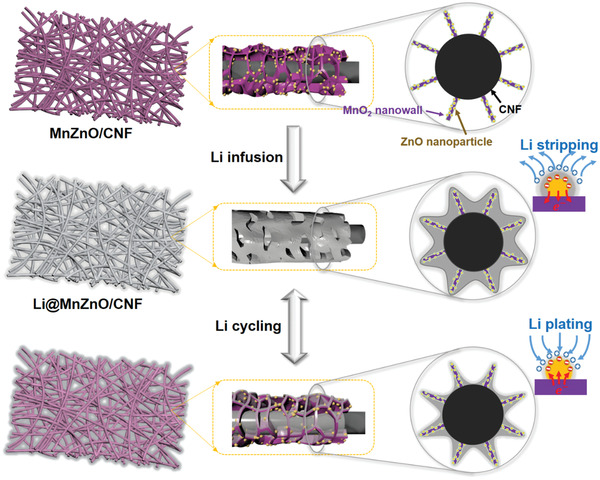
Schematic of the heterojunction with coaxial nanochamber structure design for Li trapping and reversible cycling.

Herein, nanochamber structures composed of oxide–oxide heterojunctions grown on carbon nanofiber (CNF) are proposed to improve lithium utilization and prolong the lifespan of LMA under high rates and capacities. To verify this speculation, as shown in **Figure** **1**, we herein design a MnO_2_–ZnO heterojunction (denoted MnZnO), which provides intimately bonded interfaces for fast transfer of electrons and high binding energy for strong absorption of Li‐ions. Moreover, ultrathin walls of nanochambers loaded with multiple lithiophilic sites serve as constrain boundary and stress‐buffering space for restricting lithium loading, which enables uniform Li deposition layers within submicroscale instead of blocking the original porous structure during cycling. As a result, the active sites on large lithiophilic open‐porous surfaces are effectively exposed and highly refreshable in each cycle, linking the fast electron/ion paths in the whole framework to achieve highly reversible Li plating/stripping during repeated cycling. Even under a record‐high current density of 50 mA cm^−2^, the as‐proposed Li@MnZnO/CNF electrode works stable when delivering a high reversible areal capacity of 10 mAh cm^−2^, corresponding to a lithium utilization of 70%, which is among the highest records in this field. The Li@MnZnO/CNF as anode also leads to excellent cycling stability and high capacity retention under high rates up to 5 C and 5 A g^−1^ when assembled into full cells or Li‐ion capacitors (LICs), respectively.

## 2 Results and Discussion

**Figure 2:**
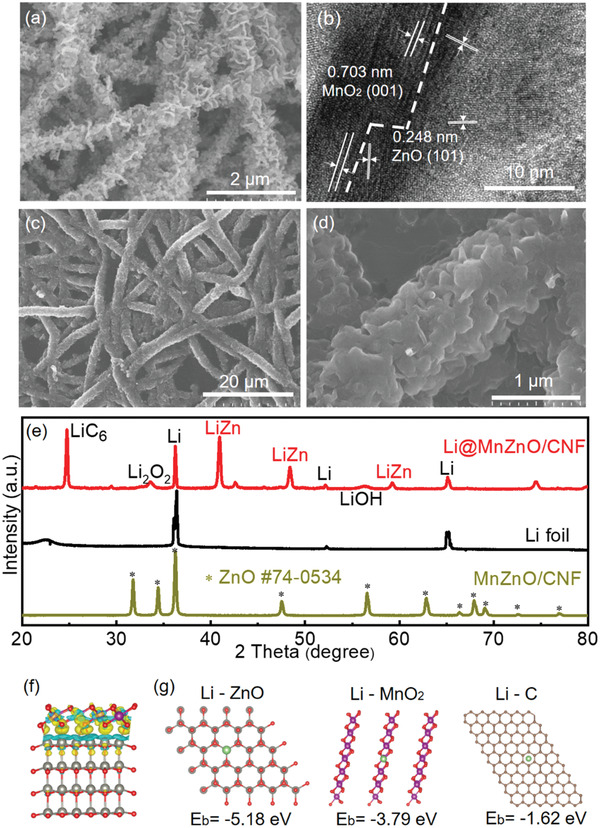
a) SEM image of MnZnO/CNF. b) HRTEM image of MnZnO/CNF. c,d) SEM images of Li@MnZnO/CNF. e) XRD patterns of the MnZnO/CNF, Li@MnZnO/CNF, and Li foil. f) Charge density difference of ZnO/MnO_2_ interface. g) The binding energy of one Li atom adsorbed on ZnO, MnO_2_, and carbon.

To fabricate the MnZnO/CNF host, ZnO nanoparticles and MnO_2_ nanowalls were grown on CNF via a carbonization process and an in situ growth strategy (see Experiment Section in Supporting Information). The in situ hydrothermal redox reaction guarantees an ultratight combination between MnO_2_ nanowalls and CNF. Notably, the as‐prepared MnZnO/CNF host retains the good mechanical flexibility of the free‐standing CNF membrane (Figure S1, Supporting Information). The morphology of MnZnO/CNF observed by scanning electron microscope (SEM) shows that MnO_2_ nanowalls are uniformly distributed and tightly attached to CNF (**Figure** **2**a). The aligned MnO_2_ nanowalls with an average thickness of 20–30 nm and height of 200–300 nmdivide each nanofiber surface into numerous nanochambers with the side length of 100–300 nm,while ZnO nanoparticles with an average size of ≈50 nm are loaded in the nanochambers (Figure 2a and Figure S2a–d, Supporting Information). The uniform distribution of MnO_2_ and ZnO on single nanofiber is further verified by energy dispersive spectrometer mapping (Figure S3, Supporting Information). The high‐resolution transmission electron microscopy observation further confirms that ZnO nanoparticles are anchored tightly on the flaky MnO_2_ nanowalls with intimately bonded interfaces to form MnO_2_–ZnO heterojunction (Figure 2b and Figure S4, Supporting Information). Herein, the MnZnO/CNF has a high specific surface area of 140 m^2^ g^−1^ and a large pore volume of 0.22 cm^3^ g^−1^ (Figure S5, Supporting Information). The abundant mesopores and slit macropores contributed from the nanochambers divided by nanowalls account for 68% of the total pore volume.

The Li@MnZnO/CNF electrode was then fabricated by a thermal infusion process. During the molten Li infusion, the fiber diameter of the Li@MnZnO/CNF increases from ≈1 to ≈2 µm because of the occupation of metallic Li in the designed chambers (Figure 2c,d). Benefiting from the trapping effect of nanochambers on Li infusion, the 3D architecture and porous surface structure were well retained in the Li@MnZnO/CNF, which is significantly different from morphology of ZnO/CNF with uncontrollable molten lithium that blocks the whole framework (Figure S6, Supporting Information). X‐ray diffraction (XRD) and X‐ray photoelectron spectroscopy (XPS) were employed to analyze the component changes before and after the molten Li infusion (Figure 2e). The XRD pattern of MnZnO/CNF host indicates the successful growth of ZnO and amorphous birnessite‐type MnO_2_ in the CNF matrix (Figure 2e and Figure S7, Supporting Information).^[^ ^26^, ^27^, ^28^, ^29^, ^30^, ^31^ ^]^ Meanwhile, the negative shift for binding energies of Zn 2p3/2 and Zn 2p1/2 peaks in XPS analysis (Figure S8, Supporting Information) suggests the potential substitution by Mn ions in ZnO lattice and formation of Zn—Mn bonding structure, forming intimately bonded interfaces.^[^ ^32^ ^]^ After the Li loading in the host, the diffraction peaks of ZnO disappear with the emergence of typical peaks of Li–Zn alloy and LiC_6_ in the XRD pattern owing to the reaction with Li at a high temperature.

Density functional theory (DFT) calculations were used to further analyze the interfacial binding of the heterojunction. A high binding energy of 0.114 eV Å^−2^ can be obtained at the MnO_2_–ZnO interface, which is much higher than vdW type interaction binding. As shown in the charge density difference in Figure 2f, the MnO_2_–ZnO interface exhibits a strong electron coupling effect, which contributes to multiple active sites for driving the electrons flow quickly. The interaction between Li and different substances of ZnO, MnO_2_, and carbon were also revealed by the DFT calculations (Figure 2g and Figure S9, Supporting Information). A high binding energy of −5.18 eV was obtained between the Li and (002) plane of ZnO, showing its stronger adsorption ability to Li compared with the pristine carbon and MnO_2_. Meanwhile, the nucleation overpotential of ZnO/CNF (4.7 mV) at 1.0 mA cm^−2^ is much lower than CNF (33.1 mV) and MnO_2_/CNF (21.4 mV), indicating that ZnO nanoparticles can serve as nucleation sites to reduce the Li nucleation barrier (Figure S10, Supporting Information). Hence, uniformly dispersed ZnO nanoparticles in the MnZnO heterojunction contribute to a nearly zero initial Li nucleation overpotential of MnZnO/CNF electrode. It can be concluded that MnZnO heterojunction provides multiple sites with strong absorption ability for Li‐ions while the intimately bonded interfaces ensure the fast transfer for electrons, leading to promoted transformation kinetic for Li‐ion plating and stripping process.

The Coulombic efficiency (CE) is considered as a key factor to evaluate the capacity reversibility with Li fully utilized and without any excessive Li supplement. When the plating capacity is 1.0 mAh cm^−2^, the CE of the MnZnO/CNF host maintains over 98.6% for 300 cycles and over 95.5% for 500 cycles at 1.0 mA cm^−2^ (Figure S11a, Supporting Information). Meanwhile, when the current density increased to 5.0 mA cm^−2^, the CE of MnZnO/CNF can remain stable at 94.5% after 120 cycles (Figure S11b, Supporting Information). Further increasing the capacity to 4.0 mAh cm^−2^, a high CE over 95.4% was obtained after the 80 cycles (Figure S11c, Supporting Information). In contrast, the CEs of ZnO/CNF and MnO_2_/CNF electrode decay much faster in all abovementioned testing condition, suggesting the severe Li consumption by dendritic growth and dead Li formation in the cells. These results reveal that MnZnO heterojunction with high Li‐ion absorption ability and rapid charge transfer paths contributes to significant improvement in reversibility for Li plating/stripping process compared with the single MnO_2_ or ZnO.

**Figure 3:**
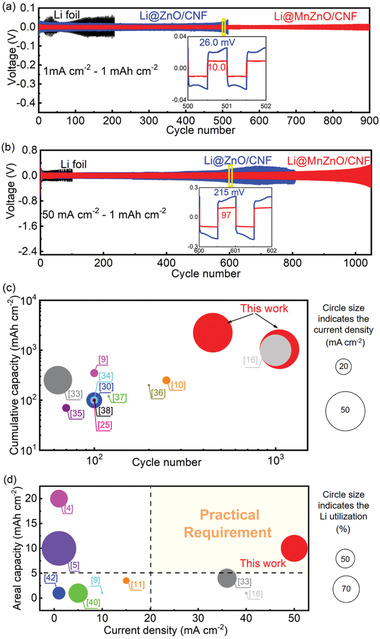
The voltage profiles of symmetrical cells with bare Li foil, Li@ZnO/CNF, and Li@MnZnO/CNF electrodes under current densities of a) 1.0 mA cm^−2^ and b) 50 mA cm^−2^ with a fixed capacity of 1.0 mAh cm^−2^. c,d) Electrochemical performance of symmetrical cells with Li@MnZnO/CNF electrode compared with other reported Li‐metal composite anodes in terms of current density, areal capacity, lithium utilization, cycling life, and the cumulative Li‐plating capacity.

Galvanostatic discharge/charge tests of the symmetric cells were performed to further verify the cycling performance of the as‐fabricated Li@MnZnO/CNF electrode. At a current density of 1.0 mA cm^−2^ and a cycling capacity of 1.0 mAh cm^−2^, the Li@MnZnO/CNF exhibits ultrastable voltage curves over 900 cycles (1800 h) and low overpotentials (10 mV) in the 500th cycle (**Figure** **3**a). However, the voltage profile of the bare Li electrode and Li@ZnO/CNF electrode show larger overpotentials and sharp fluctuations after 500 and 100 cycles, respectively, indicating the dendrite formation and the continuous side reactions between fresh lithium and electrolyte owing to the unstable electrode surface with limited exposed lithiophilic sites.

As the current density was increased to 50 mA cm^−2^, the Li@MnZnO/CNF electrode still displays flat charge/discharge profiles with a much smaller overpotential (97 mV) than that of Li@ZnO/CNF (215 mV) at 600th cycle (Figure 3b). The Li@MnZnO/CNF electrode maintains such a good performance even after 1050 cycles. In sharp contrast, the cell with Li@ZnO/CNF and bare Li foil, respectively, experiences short circuit at about 700th and 100th cycle owing to the separator rupture caused by the growth of lithium dendrites. The ultralong lifespan and cycle stability at different current densities further confirm the remarkable high‐rate performance of Li@MnZnO/CNF electrodes (Figure S12a, Supporting Information). As the current densities varies with the range of 1.0–50 mA cm^−2^, the symmetrical cell with Li@MnZnO/CNF electrode displays a stable voltage polarization with a small overpotential of 85 mV at 50 mA cm^−2^. As increasing to a capacity of 3.0 mAh cm^−2^ at a current density of 50 mA cm^−2^, the electrode worked stably for over 600 cycles with a flat voltage fluctuation (Figure S12b, Supporting Information). The electrode also demonstrates a long lifespan of 450 cycles without short circuit for a high capacity of up to 5.0 mAh cm^−2^ (Figure S12c, Supporting Information). When the capacity was further increased to 10.0 mAh cm^−2^, the electrode shows a cycling stability for over 100 cycles (Figure S12d, Supporting Information). Since the Li loading on each electrode is 14.3 mAh cm^−2^, the reversible plating/stripping capacity of 10 mAh cm^−2^ yields a high lithium utilization of 70%.

Above results demonstrate that Li@MnZnO/CNF electrode shows a long lifespan of 1050 cycles at a high current density of 50 mA cm^−2^ realized for a large cumulative plating capacity over 1050 mAh cm^−2^, which is among the highest value for previous reports (Figure 3c and Table S1, Supporting Information).^[^ ^9^, ^10^, ^16^, ^25^, ^30^, ^33^, ^34^, ^35^, ^36^, ^37^, ^38^ ^]^ It is worth pointing out that our Li@MnZnO/CNF electrode shows an excellent lithium utilization as high as 70% even under a high capacity and high current density simultaneously, comparable to the highest values in the literatures under relatively low current densities (Figure 3d and Table S2, Supporting Information).^[^ ^4^, ^5^, ^9^, ^10^, ^11^, ^16^, ^30^, ^33^, ^39^, ^40^, ^41^, ^42^ ^]^ This work is the only one that achieves high Li utilization in the shadowed domain at the top right‐hand corner of Figure 3d, showing its superior potential for practical application of high current densities of >20 mA cm^−2^ and high areal capacity of >5 mAh cm^−2^.

**Figure 4:**
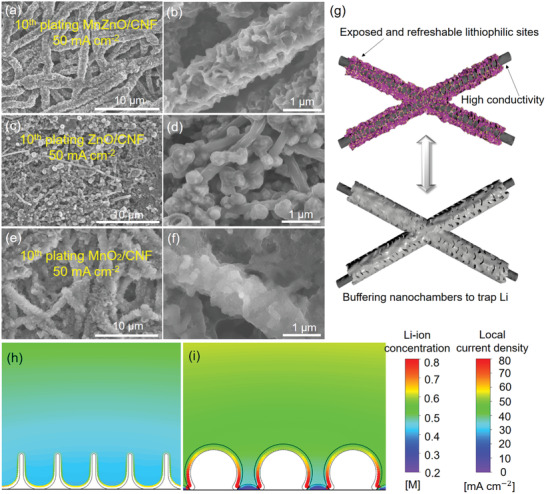
SEM images of a,b) MnZnO/CNF, c,d) ZnO/CNF, and e,f) MnO_2_/CNF after tenth plating at a current density of 50 mA cm^−2^ with a fixed capacity of 1.0 mAh cm^−2^. g) Schematic illustrations of Li deposited on the MnZnO/CNF host. Simulation results of h) MnZnO/CNF and i) ZnO/CNF. Colors in electrolyte domain denote concentration of Li‐ions, and colors on the electrode indicate the local current density. Lines at the bottom represent the initial shape of electrodes.

To further verify the guiding effect of MnZnO/CNF on Li deposition, the structural evolution was observed after plating 1.0 mAh cm^−2^ of Li on the skeletons at different current densities. After the tenth cycle of Li deposition at current densities less than 50 mA cm^−2^, the deposited Li was effectively trapped in the nanochambers on each nanofiber with relative uniform morphology, showing a high reversibility of Li plating/stripping (**Figure** **4**a,b and Figure S13a,b, Supporting Information). The same tests were carried out on ZnO/CNF host and MnO_2_/CNF host to figure out the guiding effect of ZnO and MnO_2_ separately. It can be seen that the lithium preferred to gather around ZnO nanoparticles in the ZnO/CNF scaffold at all current densities due to its high binding energy with Li‐ions (Figure 4c,d and Figure S13c,d, Supporting Information). However, the deposited Li blocked the fiber pores owing to the limited exposed lithiophilic sites and absence of deposition space restriction. On the MnO_2_/CNF scaffold, the restriction effect of nanochambers worked well to guide Li deposition at 1.0 and 10 mA cm^−2^ (Figure S13e,f, Supporting Information). Increasing the current density to 50 mA cm^−2^, massive lithium distributed disorderly between the fibers instead of being restricted in the nanochambers (Figure 4e,f), which is attributed to the weaker binding energy with Li atom for single MnO_2_.

Notably, with a high capacity of 5.0 mAh cm^−2^ at 50 mA cm^−2^, the structure of MnZnO/CNF was still well‐preserved with dendrite‐free morphology for ten times (Figure S14a, b, Supporting Information), suggesting that the high capacity and rate performance can be reserved validly in MnZnO/CNF host at the same time. It can be seen that the lithium was effectively deposited as thin layers within submicro scale under the guidance of fast electron/ion paths by the MnZnO heterojunction and confined in the nanochambers. Moreover, the macropores between nanofibers can be still maintained for promoting the diffusion of Li‐ions after repeated cycles.

The inhibited volume expansion is confirmed by cross‐sectional SEM observation of the Li@MnZnO/CNF electrode after cycling. When cycling at an ultrahigh current density of 50 mA cm^−2^ with an area capacity of 1.0 mAh cm^−2^, the volume change of Li@MnZnO/CNF is only about 4.1% after 300 cycles (Figure S15, Supporting Information). In contrast, the bare Li shows a much larger volume expansion of 51.4% after the first 100 cycles. The highly improved structural stability of Li@MnZnO/CNF than bare Li proves the importance of guiding Li‐ions deposited in a confined space. The impressive structural stability and negligible volume expansion of Li@MnZnO/CNF at ultrahigh current density are ascribed to the buffering function of the nanochambers as well as the effective guidance of MnZnO heterojunction for Li plating/stripping.

The interfacial stability and inner resistance of the symmetric cells were further analyzed by electrochemical impedance spectrum in Figure S16, Supporting Information. The interfacial resistance of Li@MnZnO/CNF is 13.3 Ω before cycling. This value decreases to 0.7 Ω after 100 cycles at 50 mA cm^−2^. The bare Li‐based symmetric cell shows a much larger initial interfacial resistance of 29.5 Ω, and this value decreases to near zero after 100 cycles owing to the accumulation of Li dendrites and the occurrence of an internal short circuit. The much lower interfacial resistance suggests the stable electrode interface and superior Li stripping/deposition kinetics of the Li@MnZnO/CNF electrode with favorable nanochamber‐structured MnZnO heterojunction. Thus, it ensures a highly effective and reversible charge/discharge process, improving the lithium utilization without Li dendrite growth and dead Li formation.

To further visualize the Li deposition behaviors on different scaffold structure, we simulated cells working under an average current density of 50 mA cm^−2^ (details see Figure S17, Supporting Information). We compare the Li‐ion concentration and local current densities for the two different electrodes in Figure 4h,i. The MnZnO/CNF electrode exhibits uniform local current density on the electrode and Li‐ion distribution without obvious gradient. Therefore, the Li‐ions and electrons can be transported smoothly in the nanochambers. The shortened electron/ion transport paths with boundary restriction ensure the controllable Li deposition with high reversibility. In contrast, because of the lithiophilicity difference between ZnO and CNF in the ZnO/CNF, Li‐ions prefer to deposit on ZnO, resulting in larger particle size and significantly different local current densities. Moreover, an obvious Li‐ion concentration gradient builds up in the narrow gaps near the CNF. The lowest concentration decreases to 45% of that at the top of ZnO. Therefore, hot spots with high ionic flux are formed around the ZnO particles, while few Li‐ions can be transported to the CNF surface. The blocked pores would promote the formation of Li dendrite and dead Li in the follow‐up plating/stripping process because of the nonuniform Li deposition.

Based on above analysis, we deem that the highly reversible Li plating/stripping behaviors in MnZnO/CNF is due to its fast electron/ion transport paths contributed from confined deposition space and refreshable lithiophilic active sites with large exposed surface area (Figure 4g). The lithiophilic nanochamber‐structured heterojunction with intimately bonded interfaces grown on the conductive scaffold constructs interlinked channels and open pores for fast electron/ion transport paths during Li deposition. Moreover, the deposited Li can spread over as thin layer in the designed confined nanochambers under the effective guidance of MnZnO heterojunction with expanded lithiophilic surface area. Thus, it contributes to high reversibility of Li plating/stripping behaviors and renewal of active lithiophilic sites during every cycle, which is beneficial for high lithium utilization and excellent cycling performances under ultrahigh current density.

**Figure 5:**
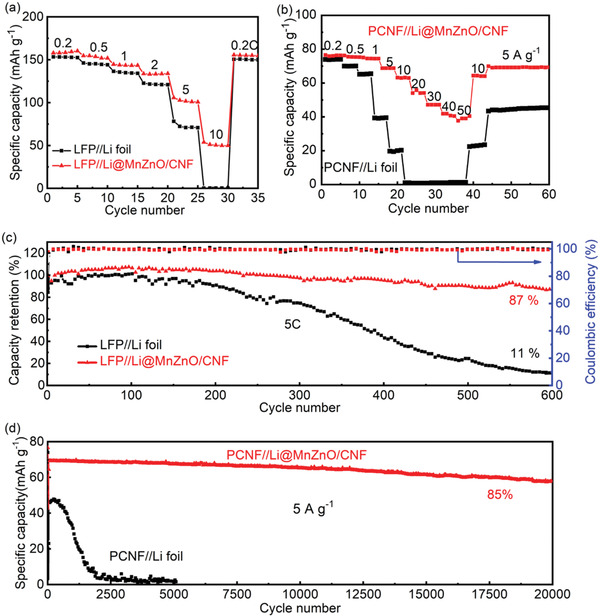
Electrochemical performance of Li@MnZnO/CNF and Li foil‐based full cells and LIC. The rate performance of a) LFP full cells and b) LICs. The cycling performance of c) LFP full cells at 5 C and d) LICs at 5 A g^−1^.

To further evaluate the prospect of Li@MnZnO/CNF anodes in practical battery systems, full cells with LiFePO_4_ (LFP) as cathode were assembled for electrochemical tests. As shown in **Figure** **5**a, the LFP//Li@MnZnO/CNF full cell delivers a discharge capacity up to 51 mAh g^−1^ under 10 C, while the discharge capacity of the LFP//Li foil cell at the same rate is almost negligible. The voltage–capacity curve shows that the full cell with Li@MnZnO/CNF anode has a much smaller and more stable polarization than that of bare Li (70.3 vs 94.6 mV), benefiting from the smaller impedance and more stable SEI (Figure S18a, Supporting Information). Under a high charge/discharge rate of 5 C, LFP//Li@MnZnO/CNF cell shows good cycling stability with a high capacity retention of 87%, while the specific capacity of LFP//bare Li full cell decreases to 11% after 600 cycles (Figure 5c). Besides, due to the fast kinetics characteristics and stable interface, such excellent rate performance was also achieved when the Li@MnZnO/CNF was used as anode for a LIC with porous carbon nanofibers cathode. As shown in Figure 5b, the LIC delivers a high discharge capacity of 40.5 mAh g^−1^ under an ultrahigh rate of 50 A g^−1^, showing a 52.7% retention of the initial capacity. Moreover, a capacity retention of 85% at 5 A g^−1^ was obtained after 20 000 cycles (Figure 5d). In contrast, the LIC with bare Li as anode shows nearly no capacity at 20 A g^−1^ and fast decay during cycling process. Moreover, the Li@MnZnO/CNF anode also endows the full cell with LiNi_0.8_Mn_0.1_Co_0.1_O_2_ (NCM811) cathode with a high specific capacity of 187 mAh g^−1^ and high capacity retention of 82% after 300 cycles at 1 C (Figure S20, Supporting Information). The superior performance further confirms that Li@MnZnO/CNF is a promising LMA for high‐rate and long‐lifespan Li metal batteries with high lithium utilization.

## 3 Conclusion

In summary, we reported a design of MnO_2_–ZnO heterojunction with nanochamber structure coaxially grown on the interlinked highly conductive CNF framework as Li host for long‐lifespan and high‐rate LMAs with high lithium utilization. The MnZnO heterojunction with intimately bonded interfaces is tightly anchored on the CNF to build fast charge‐transferring bridges. The uniformly dispersed superlithiophilic sites on the open‐porous nanochambers increase the available nucleation sites on the chamber surfaces, facilitating uniform Li deposition for spreading over within submicro scale even under high capacities. Thus, reversible plating/stripping process and renewable active sites can be guided by fast electron/ion transport paths in the nanochambers that serve as Li‐ion traps and divided buffering space. Remarkably, symmetric cells of Li@MnZnO/CNF show a high lithium utilization of 70% even at 50 mA cm^−2^ with a high areal capacity of 10 mAh cm^−2^. The Li@MnZnO/CNF as anode also contributes to a high capacity retention of 87% at 5 C after 600 cycles and 85% at 5 A g^−1^ after 20 000 cycles in LFP full cells and LICs, respectively. This work demonstrates an avenue of improving lithium utilization by effectively rectifying reversible Li deposition/stripping on an oxide–oxide heterojunction with coaxial nanochamber structure, which paves alternative ways for practical LMAs and is also expected to be expanded to the design of other alkali metal anodes.

## Conflict of Interest

The authors declare no conflict of interest.

## Supporting information

Supporting Information

## Data Availability

Research data are not shared.
