# Supplementary material for: Deeply Cyclable and Ultrahigh‐Rate Lithium Metal Anodes Enabled by Coaxial Nanochamber Heterojunction on Carbon Nanofibers
Source: Adv Sci (Weinh). 2021 Oct 23;8(23):2101940. doi: 10.1002/advs.202101940 (PMC8655213; doi:10.1002/advs.202101940)
Supplement: Supplementary file 1 — Supporting Information [file ADVS-8-2101940-s001.pdf]

© 2021 Wiley-VCH GmbH

ADVANCED SCIENCE
Open Access

Supporting Information

for *Adv. Sci.*, DOI: 10.1002/advs.202101940

Deeply Cyclable and Ultrahigh-Rate Lithium Metal Anodes Enabled by Coaxial Nanochamber Heterojunction on Carbon Nanofibers

TrungHieu Le[a], Ciqing Yang[a], Wei Lv[b*], Qinghua Liang[c], Xiehe Huang[a], Feiyu Kang[d], Ying Yang[a**]*

Supporting Information

Deeply Cyclable and Ultrahigh-Rate Lithium Metal Anodes Enabled by Coaxial Nanochamber Heterojunction on Carbon Nanofibers

TrungHieu Le[a], Ciqing Yang[a*], Wei Lv[b*], Qinghua Liang[c], Xiehe Huang[a], Feiyu Kang[d], Ying Yang[a**]

[a] State Key Laboratory of Control and Simulation of Power System and Generation Equipments, Tsinghua University, Beijing 100084, China

[b] Shenzhen Key Laboratory for Graphene-Based Materials, Engineering Laboratory for Functionalized Carbon Materials, Tsinghua Shenzhen International Graduate School, Tsinghua University, Shenzhen 518055, China

[c] Department of Chemical Engineering, The University of Melbourne, Parkville, VIC 3010, Australia

[d] Laboratory of Advanced Materials, Department of Materials Science and Engineering, Tsinghua University, Beijing 100084, China

[*] TrungHieu Le, Ciqing Yang and Wei Lv contributed equally to this work.

[**]Corresponding author:

Email address: yingyang@tsinghua.edu.cn

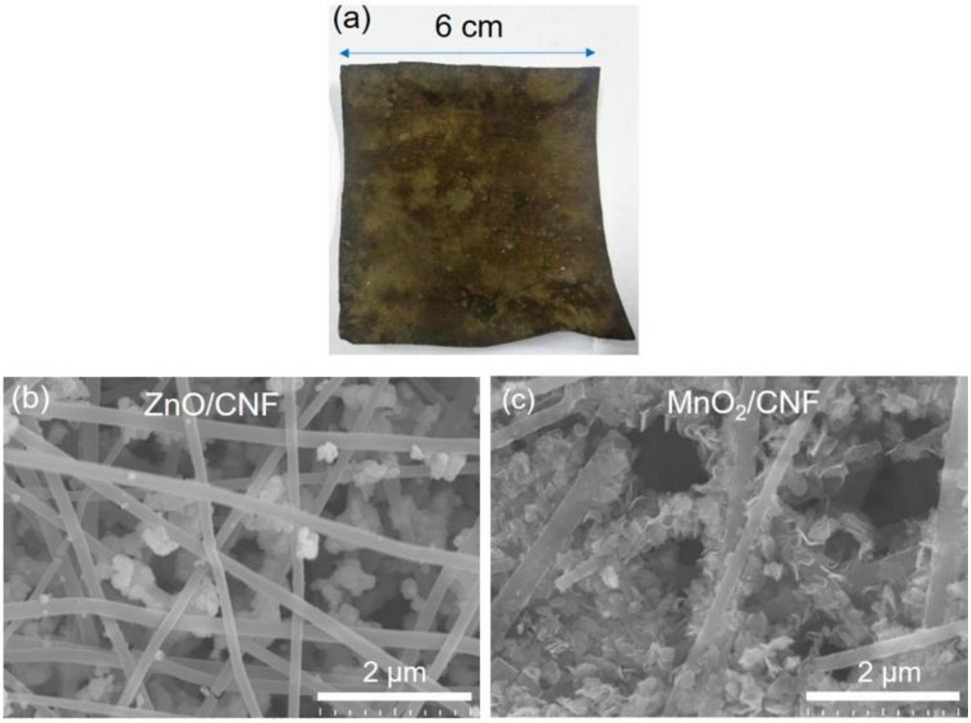

Figure S1. (a) digital photos of MnZnO/CNF; (b) SEM of ZnO/CNF; (c) SEM of MnO_2/CNF

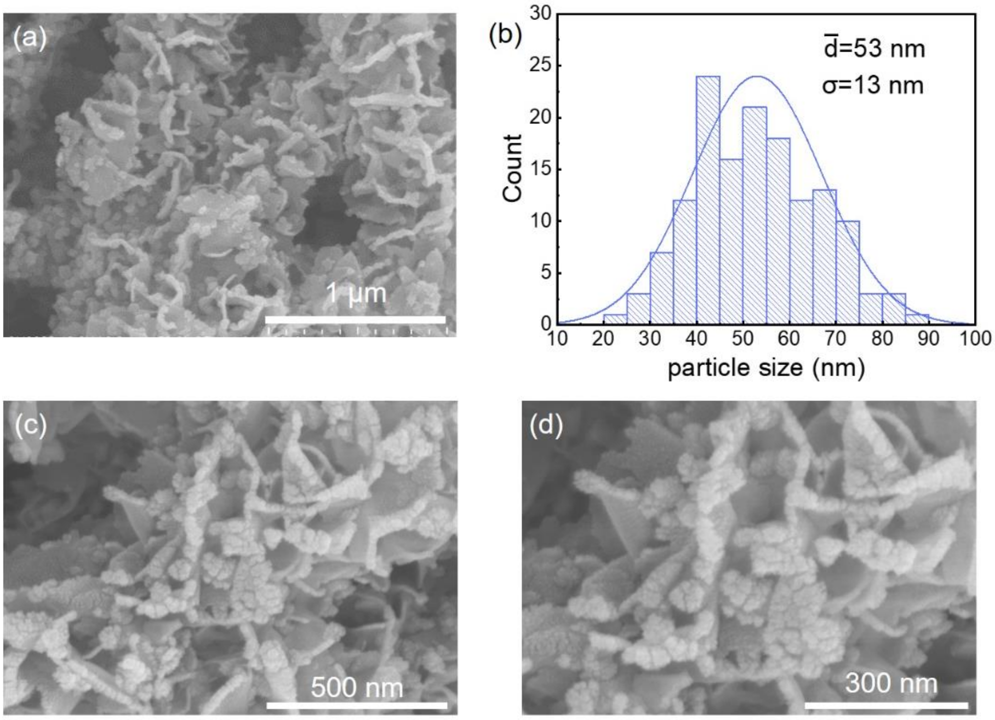

Figure S2. (a, c, d) SEM images of MnZnO/CNF; (b) particle size distribution of ZnO nanoparticles in MnZnO/CNF (data counted from Figure S2a).

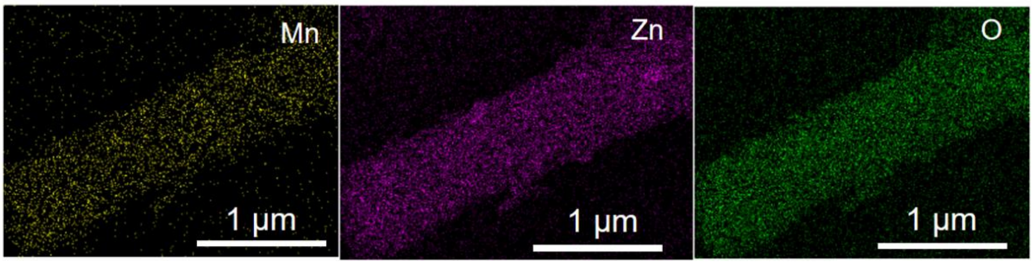

Figure S3. EDS of the MnZnO/CNF

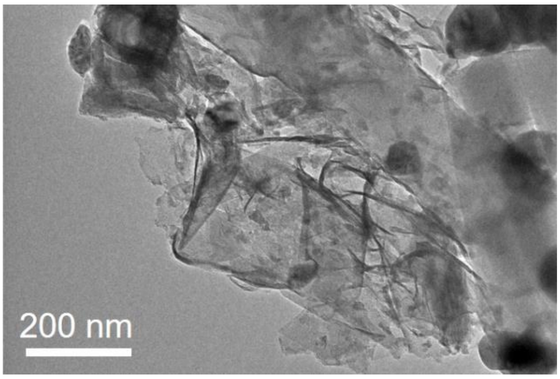

Figure S4. TEM image of MnZnO/CNF

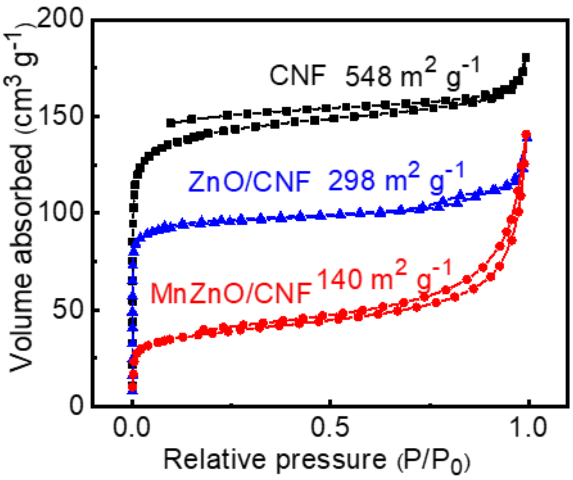

Figure S5. N_2 adsorption/desorption isotherms of CNF, ZnO/CNF and MnZnO/CNF.

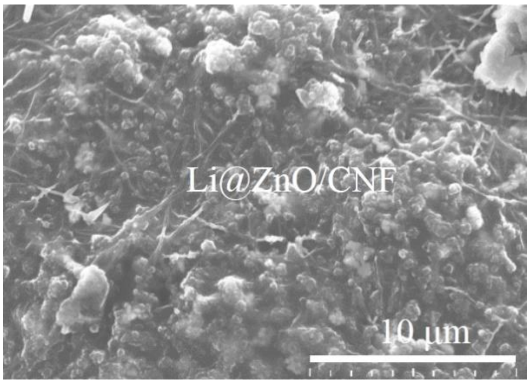

Figure S6. SEM of Li@ZnO/CNF.

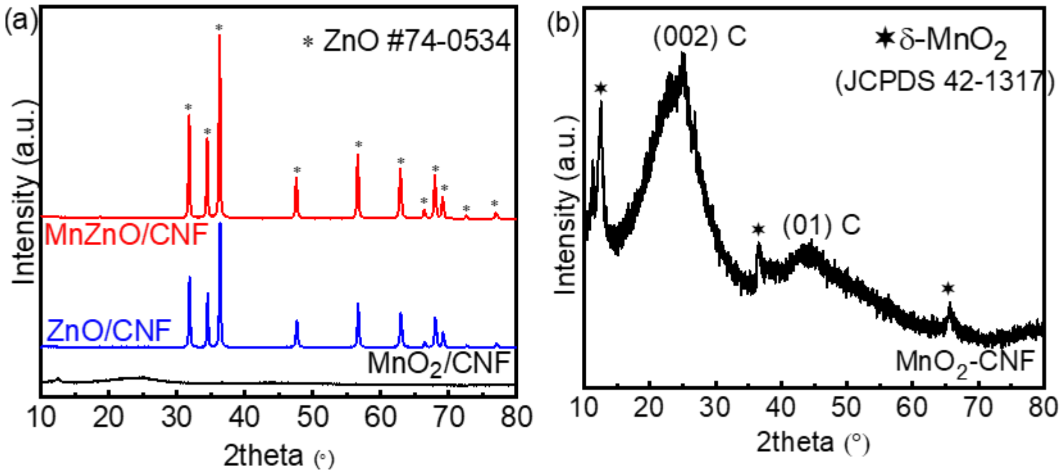

Figure S7. (a) XRD results of ZnO/CNF and MnZnO/CNF; (b) XRD results of MnO_2/CNF.

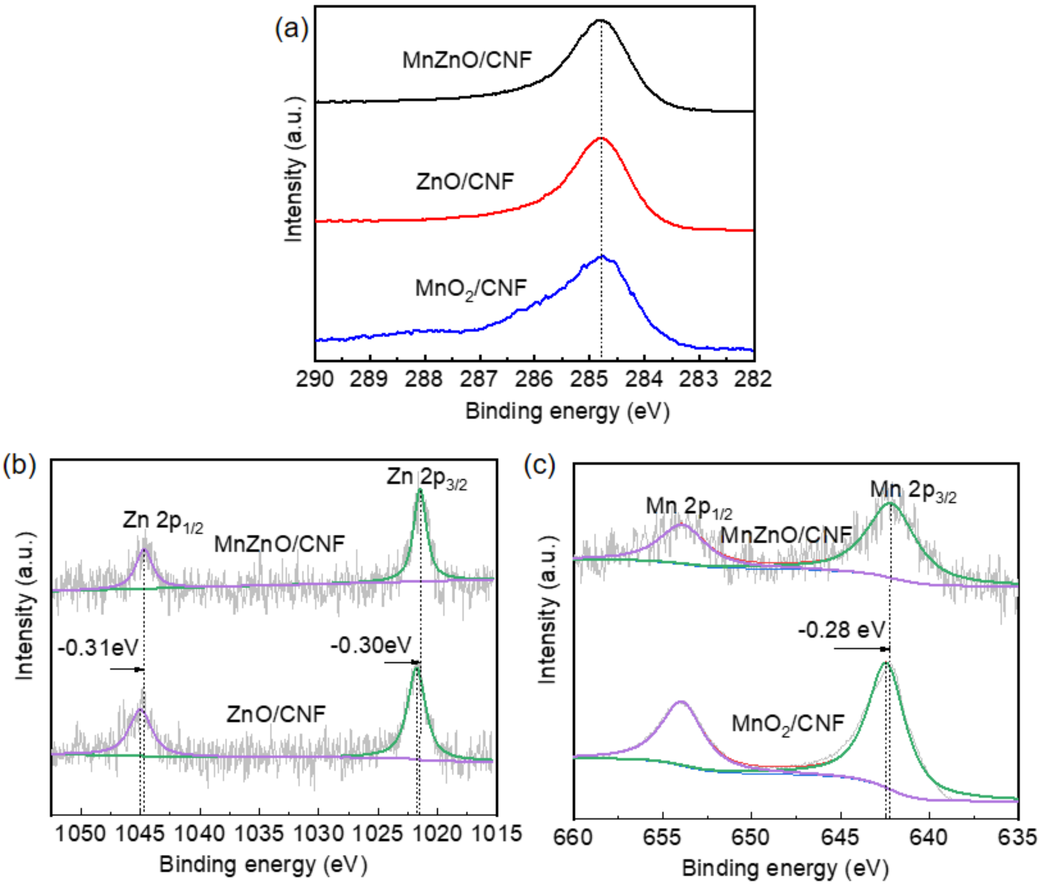

Figure S8. XPS profiles of (a) C 1s, (b) Zn 2p and (c) Mn 2p peaks of MnO/CNF, ZnO/CNF and MnZnO/CNF

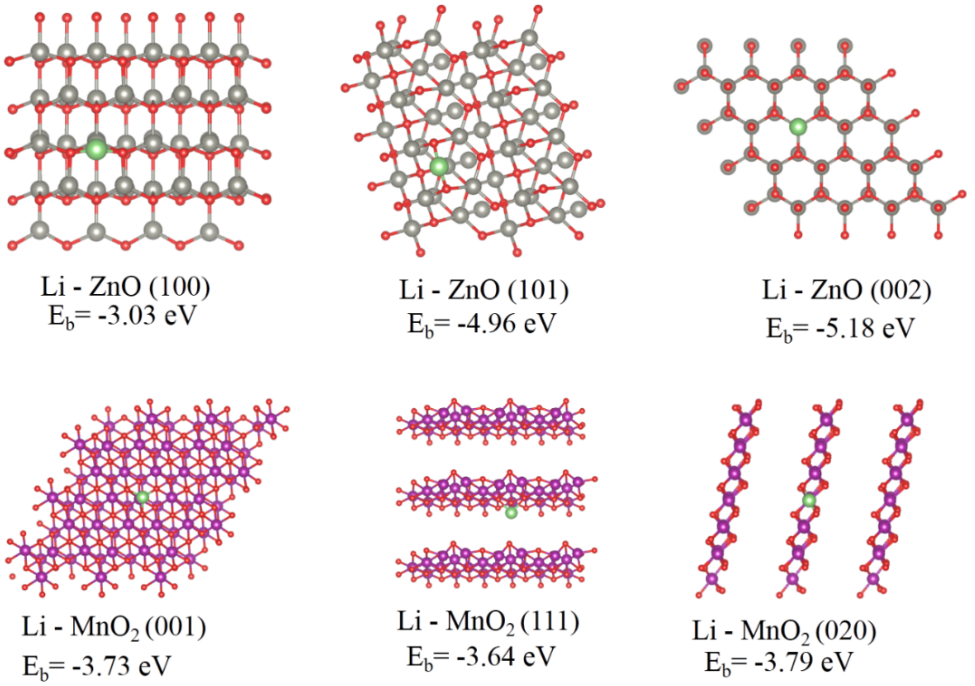

Figure S9. The binding energy of Li and different planes of ZnO, MnO_2 and C.

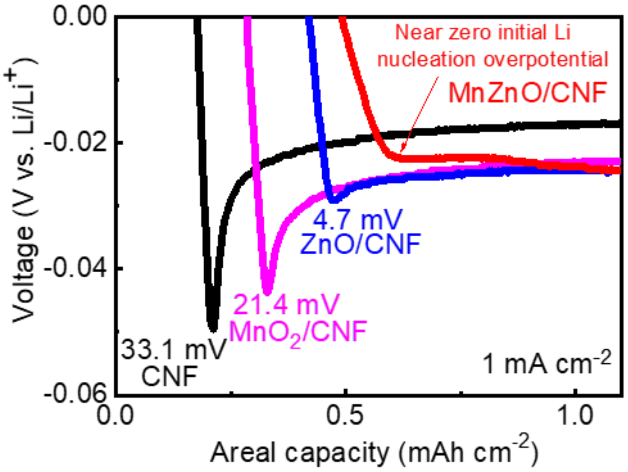

Figure S10. Voltage profiles during initial Li deposition at 1.0 mA cm^{-2} on CNF, ZnO/CNF, MnO_2/CNF and MnZnO/CNF electrodes.

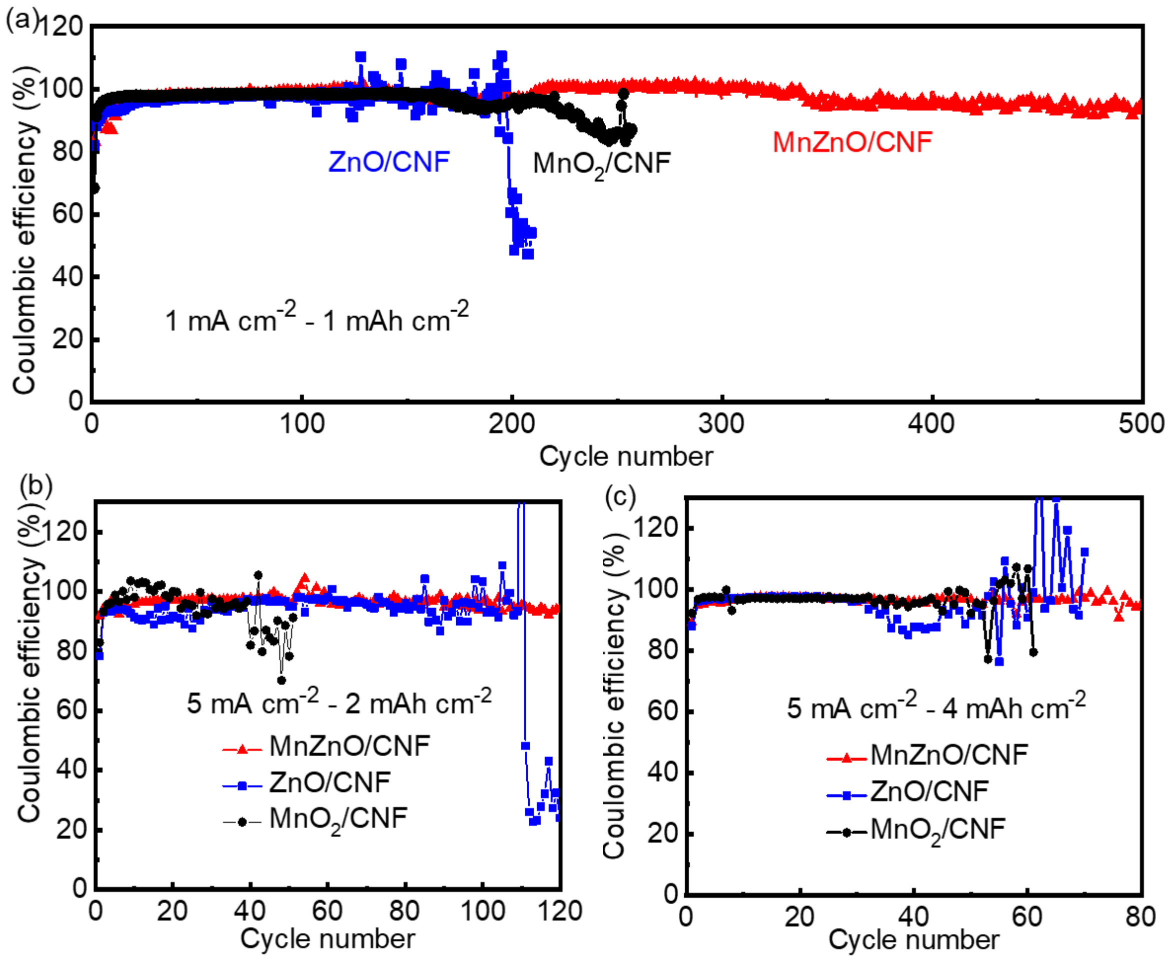

Figure S11. Coulombic efficiency of Li deposition on the MnO_2/CNF, ZnO/CNF and MnZnO/CNF substrates (a) at a current density of 1.0 mA cm^{-2} with a capacity of 1.0 mAh cm^{-2}; and at a current density of 5 mA cm^{-2} with a capacity of (b) 2 mAh cm^{-2} and (c) 4 mAh cm^{-2}.

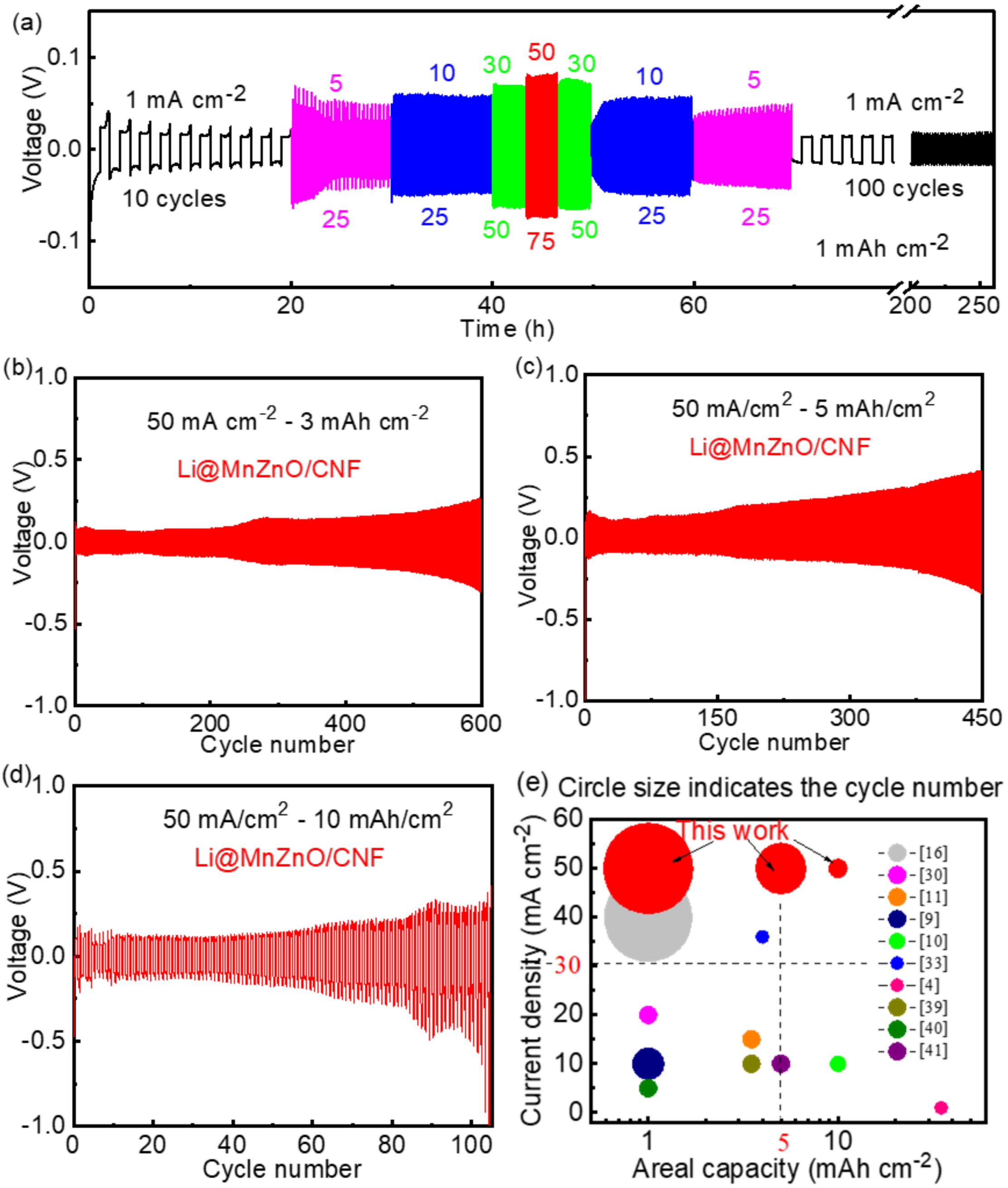

Figure S12. (a) Rate performance of the symmetrical cells with Li@MnZnO/CNF electrode; Cycling performance of symmetrical cells using Li@MnZnO/CNF composite anode under a current density of 50 mA cm^{-2} with a capacity of (b) 3 mAh cm^{-2}, (c) 5 mAh cm^{-2} and (d) 10 mAh cm^{-2}; (e) Electrochemical performance of symmetrical cells with Li@MnZnO/CNF electrode compared with other reported Li-metal composite anodes.

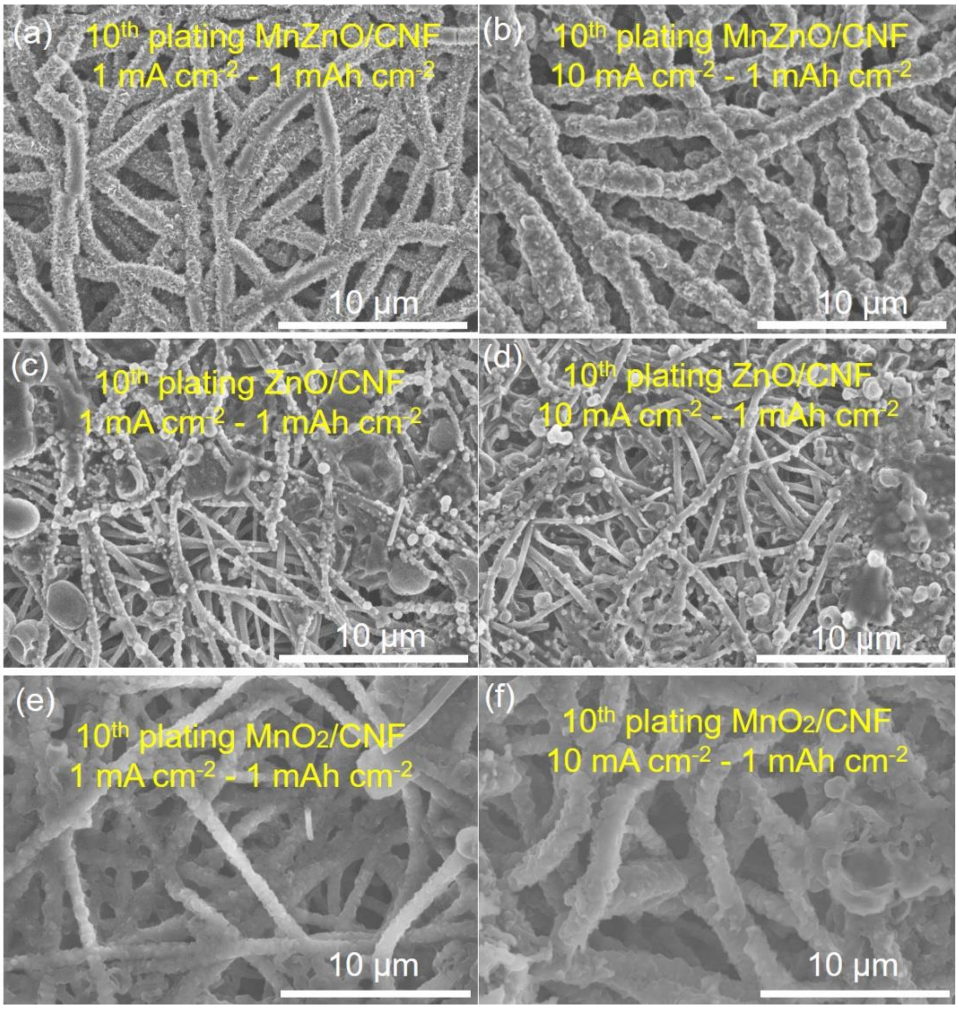

Figure S13. SEM images of 10th plating morphology of MnZnO/CNF with a fixed capacity of 1.0 mAh cm^{-2} at a current density of (a) 1.0 mA cm^{-2} and (b)10 mA cm^{-2}. SEM images of 10th plating morphology of ZnO/CNF with a fixed capacity of 1.0 mAh cm^{-2} at a current density of (c) 1.0 mA cm^{-2} and (d) 10 mA cm^{-2}. SEM images of 10th plating morphology of MnO_2/CNF with a fixed capacity of 1.0 mAh cm^{-2} at a current density of (c) 1.0 mA cm^{-2} and (d) 10 mA cm^{-2}.

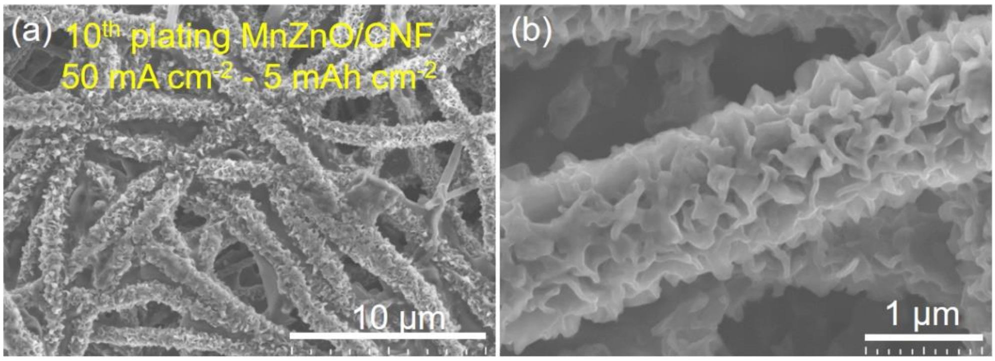

Figure S14. SEM images of 10th plating morphology of MnZnO/CNF with a capacity of 5 mAh cm^{-2} at a current density of 50 mA cm^{-2}.

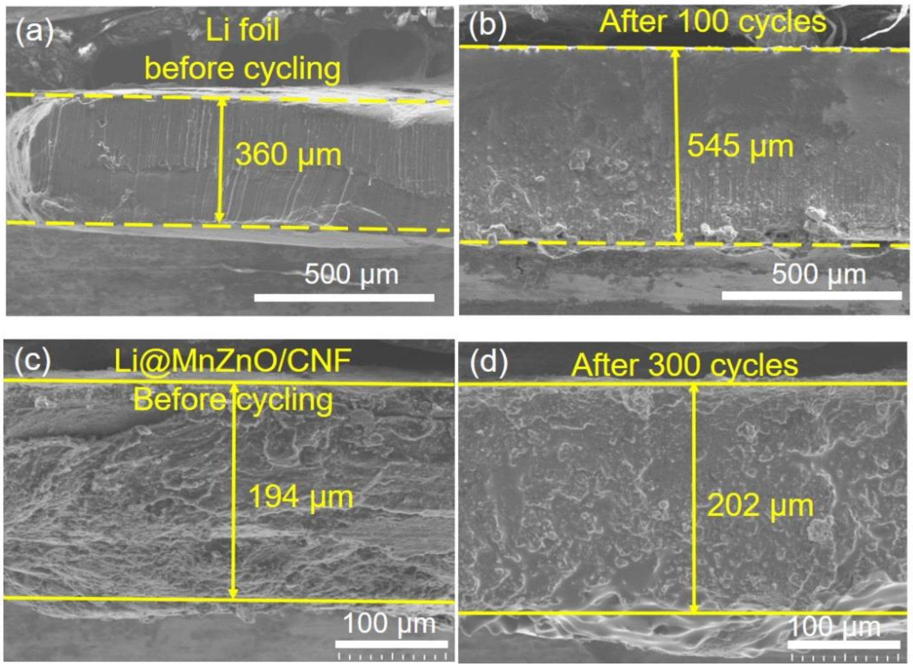

Figure S15. Cross-sectional SEM images demonstrating thicknesses and inner structures of (a) Li foil before cycling and b) Li foil after 100 cycles; c) Li@MnZnO/CNF electrode before cycling and d) and Li@MnZnO/CNF electrode after 300 cycles at a current density of 50 mA cm^{-2} under areal capacity of 1 mAh cm^{-2}.

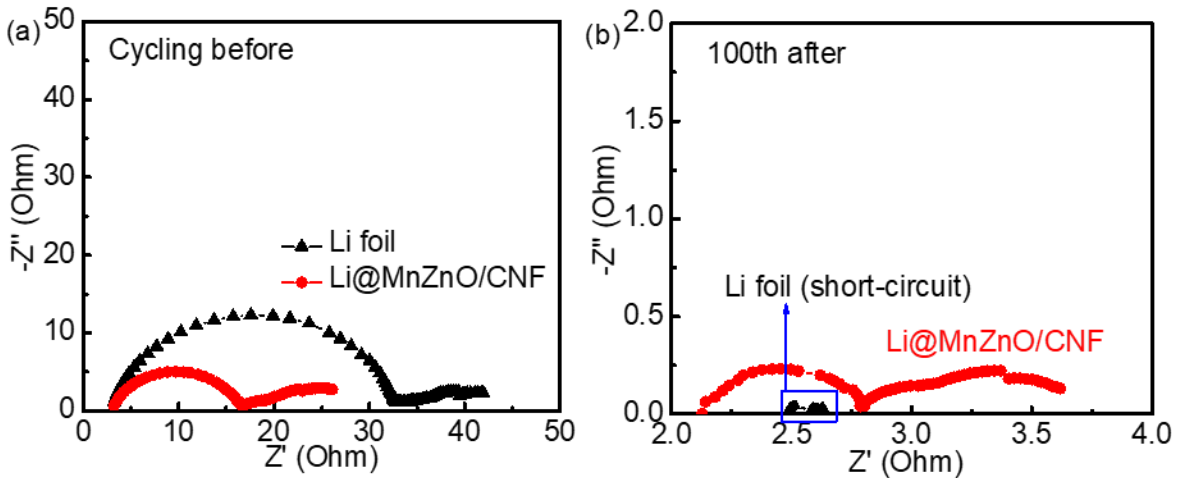

Figure S16. EIS for bare Li foil and Li@MnZnO/CNF symmetrical cell (a) before cycling and (b) after 100 cycles.

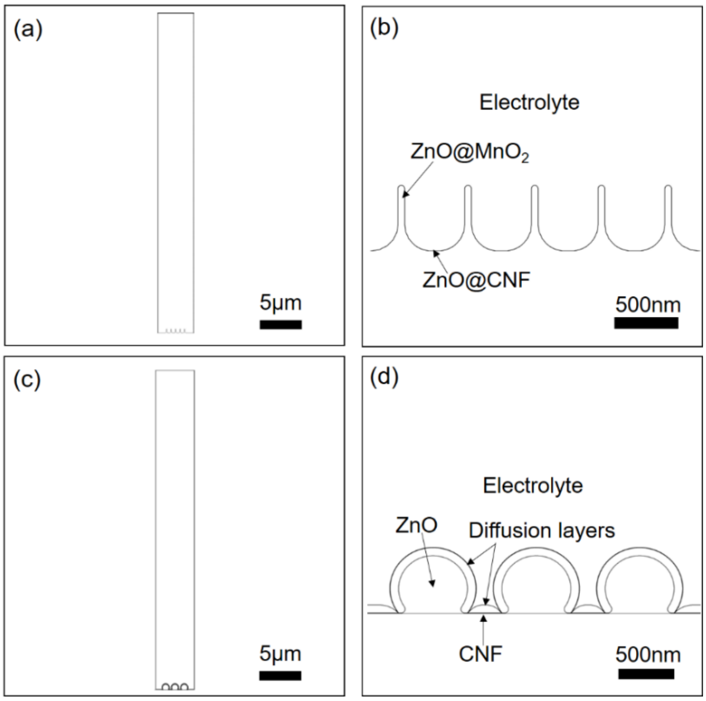

Figure S17. The simulated cell geometries for (a,b) MnZnO/CNF and (c,d) ZnO/CNF.

Electrochemical models were created as shown in above geometries. The large rectangle is the electrolyte domain with a height of 40 μm, which is corresponded with the separator thickness. In the MnZnO/CNF, MnO_2 nanowalls were 50 nm in width and 500 nm in height. In the ZnO/CNF, ZnO nanoparticles were 400nm in size. The electronic conductivity of oxides (MnO_2 and ZnO) was set to 0.01 S m^{-1}. The diffusion coefficient in the electrolyte domain was set to 10^{-10} m^2 s^{-1}. In order to consider the lithiophilicity difference between ZnO and CNF in the ZnO/CNF electrode, 100 nm thick diffusion layers were formed on the ZnO paticles and CNF with diffusion coefficients of 10^{-10} and 10^{-13} m^2/s respectively. The lithiophilicity difference is almost negligible in MnZnO/CNF, due to the evenly-dispersed ZnO lithiophilic sites in the nanochambers and on the MnO_2 nanowalls. Therefore, no diffusion layers were applied in the MnZnO/CNF electrode. The potential from the lithium metal counter electrode was set to 100 mV vs. Li/Li^+, and the average current density was set to 50 mA cm^{-2} at the bottom working electrode. The initial Li-ion concentration was 1 M. And the plots in Figures 4h and 4i were both taken from 0.5 s of the simulations.

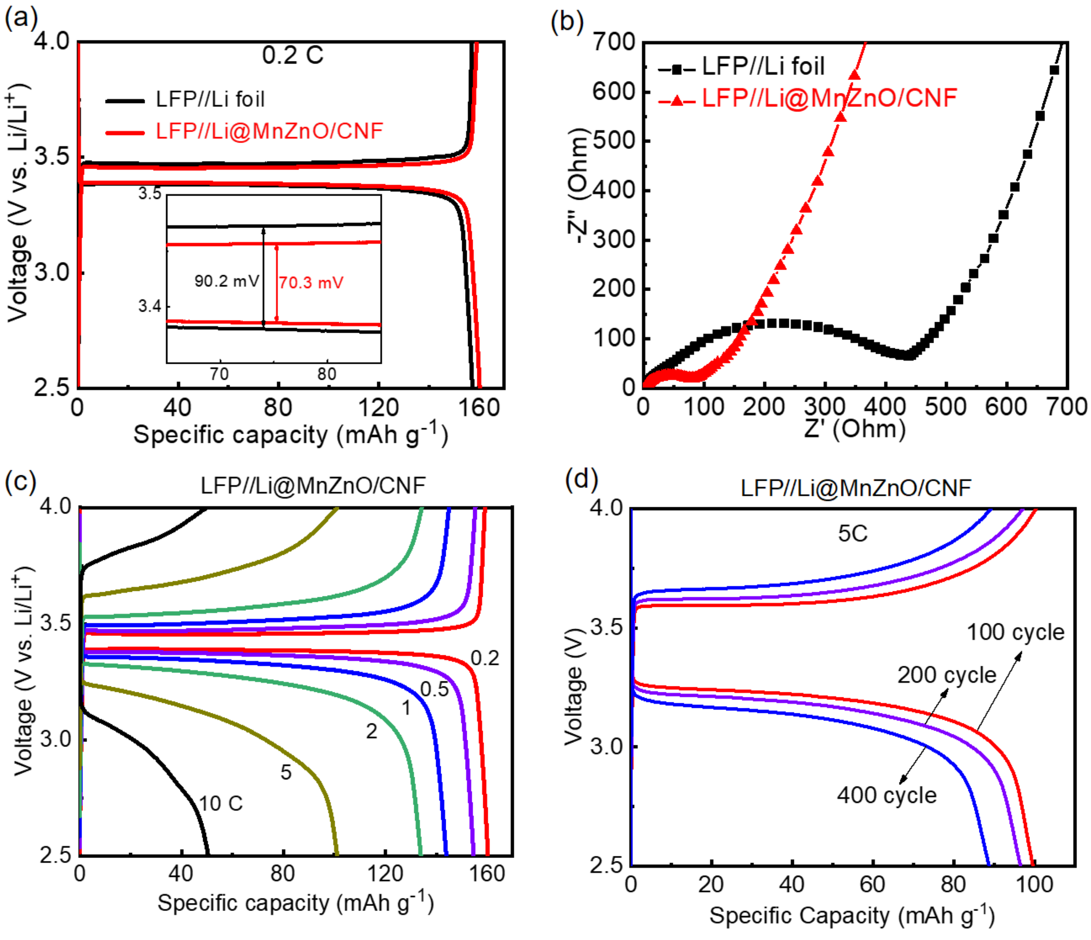

Figure S18. (a) charge/discharge profiles at 0.2 C of LFP cells. inset: enlarged profiles exhibiting the polarization; (b) EIS results for LFP cells; (c) charge/discharge profiles for rate test of LFP cells; (d) charge/discharge profiles when cycling at 5 C of LFP cells.

The EIS tests show that LFP//Li@MnZnO/CNF cell has much lower interfacial resistance (~90 Ω) than that of LFP//Li foil cell (460 Ω), resulting in improved cycling performance and stability at high rates (Figure S18b). Moreover, the LFP//Li@MnZnO/CNF cell exhibits stable voltage profiles and reversible charge/discharge capacities under different rates (Figure S18c). It also displays stable charge/discharge platforms with slight capacity degradation or polarization increase under a high rate of 5 C (Figure S18d).

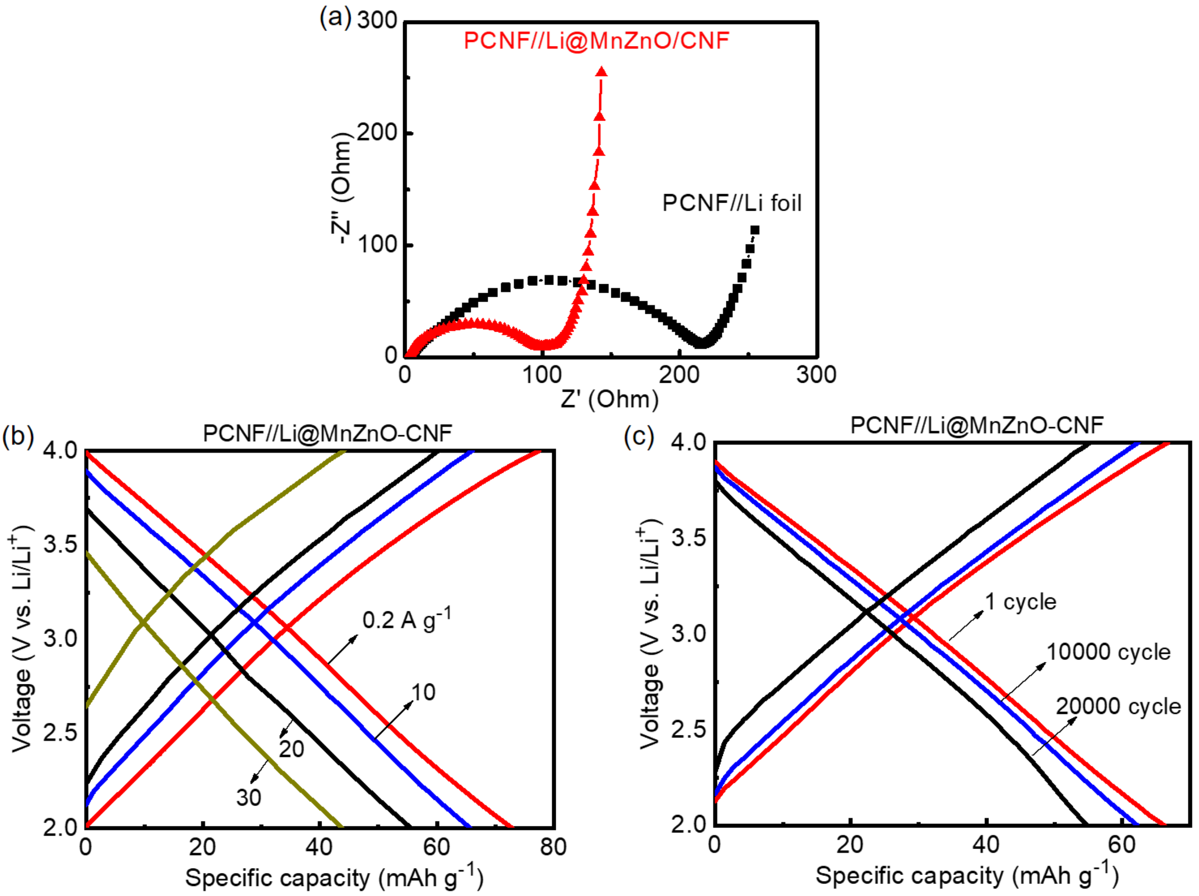

Figure S19. (a) EIS results for LICs; (b) charge/discharge profiles for rate test of LICs; (c) charge/discharge profiles when cycling at 5 A g^{-1} of LICs.

The EIS results further prove the much smaller interfacial resistance of PCNF//Li@MnZnO/CNF (102 Ω) compared with that of PCNF//Li foil (220 Ω), suggesting that the superior reaction kinetics of Li@MnZnO/CNF anode (Figure S19a). Benefiting from the superior Li-ion kinetics and excellent interfacial stability on the Li@MnZnO/CNF anode, PCNF//Li@MnZnO/CNF cell also exhibits stable voltage curves and reversible capacities in rate test and long-term cycling stability at 5 A g^{-1} (Figure S19 b, c).

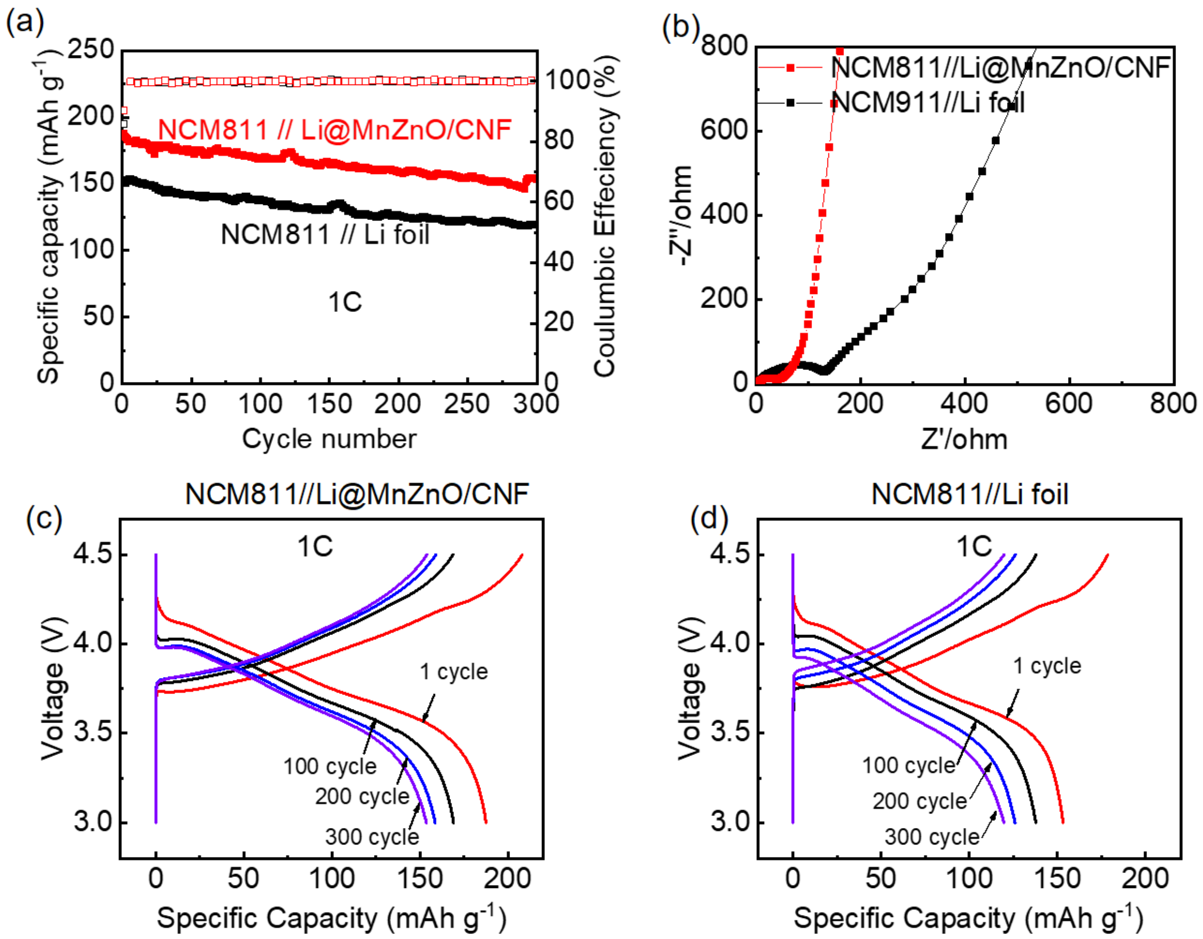

Figure S20. (a) The cycling performance of NCM811 full cells; (b) EIS results for NCM811 full cells; (c) charge/discharge profiles of NCM811 full cells with Li@MnZnO/CNF anode; (d) charge/discharge profiles of NCM811 full cells with Li foil anode.

Benefiting from the fast charge transfer and Li-ion kinetics, the NCM811 full cell with Li@MnZnO/CNF anode shows small and stable voltage polarization in 300 cycles (Figure S20 c). However, the NCM811 full cell with Li foil anode shows a sharp potential spike at the first charging process and serious polarization increase during cycling, indicating the unstable electrode interface with higher impedance (Figure S20 d).

Table S1: Comparison of the cycle life of symmetrical cells using Li@MnZnO/CNF composite anodes and various reported Li metal-based composite anodes under various current densities.

Composite anode	Current density (mA cm^{-2})	Cycling numbers	Capacity (mAh cm^{-2})	Overpotential (mV)	Ref
Silver Nanowire-Graphene	40	1000	1	~120	[16]
3D Nanoporous Nitrogen-Doped Graphene	36	63	4	~750	[33]
Li/Al_4Li_9-LiF nanocomposite	20	100	1	400	[30]
3D PMF/Li composite anode	10	250	1	~250	[9]
Silver-coated carbon fiber network	10	160	10	~45	[10]
Nanoporous Li_xSi-Li_2O composite	10	100	1	~200	[34]
Porous graphene scaffold	10	70	1	~200	[35]
Lithium-coated polymeric matrix	5	100	1	~110	[25]
TiC/C core/shell nanowire arrays	3	200	1	~85	[36]
3D oxidized polyacrylonitrile nanofiber network	3	120	1	~75	[37]
Layered reduced graphene oxide	3	100	1	~80	[38]
Li@MnZnO/CNF	50	1050	1	~100	This work
	50	450	5	~380	

Table S2. Comparison of the cycle life of symmetrical cells using Li@MnZnO/CNF composite anodes and various reported Li metal-based composite anodes under various current densities and capacities.

Composite anode	Current density (mA cm^{-2})	Cycling numbers	Capacity (mAh cm^{-2})	Li utilization (%)	Ref
Silver Nanowire-Graphene	40	1000	1	8.3	[16]
Li/Al_4Li_9-LiF nanocomposite	20	100	1	//	[30]
MgO@Wood derived carbon	15	100	3.5	17.5	[11]
3D PMF/Li composite anode	10	250	1	5.0	[9]
Silver-coated carbon fiber	10	80	10	//	[10]
3D Nanoporous Nitrogen-Doped Graphene	36	63	4	50.8	[33]
Parallelly aligned MXene-lithium	1	11	20	46.5	[4]
Li@Al_2O_3/Ni/Au	10	135	3.5	//	[39]
Copper nanowires@ cellulose nanofibers	5	100	1	50.0	[40]
Polypyrrole-Graphene Oxide	10	100	5	//	[41]
TiN/Carbon nanofiber	1	300	1	33.3	[42]
Covalently connected graphite microtubes	1	150	10	91.0	[5]
Li@MnZnO/CNF	50	1050	1		This work
	50	450	5		
	50	100	10	70.0	

Experiment Section

1. Material Synthesis

1.1 Synthesis of Polyimide (PI)

p-phenylenediamine (PPD, >99.0%; Mw = 108.14 g mol^{-1}) and 3,3',4,4'-Biphenyl tetracarboxylic diandhydride (BPDA, >99.5%; Mw = 294.22 g mol^{-1}) with the molar ratio of 1:1 were dissolved in N,N-dimethylformamide (DMF) . Afterwards, the mixture was stirred at 0°C for 24h to obtain the electrospinning solution containing 10wt% PPD and BPDA. The electrospinning process was operated with applied voltage of ~20 kV, tip-to-collector distance of 20 cm and flow rate of 1.0 mL h^{-1}. Then the PI membranes were obtained by the thermal imidization of the as-prepared electrospun fibrous membranes in a horizontal tubular furnace at 350 °C for 30 min under air atmosphere, with a heating rate of 3 °C min^{-1}.

1.2 Synthesis of ZnO/CNF

The PI membranes were then treated by plasma in the Schwarze Plasma Cleaning Machine for 2 min. 0.1 g PI was soaked in 10 g $Zn(CH_3COO)_2$ water solution (10 wt%) at 60 °C for 4 h and naturally dried for 20 h. Afterwards, PI membranes loaded with acetate precursor were carbonized in 800 °C for 1 h under Ar atmosphere with a heating rate of 10 °C/min. Finally, ZnO/CNF films were obtained after naturally cooling to room temperature with protection of Ar flow. The pure PI membranes were carbonized with same procedure to obtain CNF for comparison.

1.3 Synthesis of MnZnO/CNF

120 mg ZnO/CNF membrane was immersed in 400 mL $KMnO_4$ solution (containing 20 mg $KMnO_4$). The reaction was kept at 70 °C until the solution color changed from purple to light brown. The membrane was rinsed with deionized water several times, and then treated by vacuum drying at 100 °C to obtain MnZnO/CNF.

1.4 Synthesis of porous carbon nanofiber (PCNF)

The electrospinning solution was prepared with PPD + BPDA, and polyvinyl pyrrolidones (PVP, Mw = 30,000 g mol^{-1}) with a mass ratio of 1:1 ((PPD+BPDA) : PVP) in DMF. The parameters of electrospinning process, imidization and carbonization are the same as described above.

2. Characterization

The morphologies and elemental distributions of samples were investigated using scanning electron microscope (SEM, SU-8010) coupled with an Energy dispersive spectrometer (EDS) attachment. Transmission electron microscope (TEM, JEOL 2100F) tests were matched with SEM results to provide more details about the structure and composition of the samples. X-ray diffraction (XRD) patterns of samples were examined using D/max-2500/PC with filtered Cu Kα radiation (scanning rate: 5° min^{-1}, 2θ range: 10° ~ 80°). The specific surface areas, pore size distributions and pore volumes of the samples were calculated from the adsorption/desorption isotherms of N_2 at 77K by an automatic adsorption system (Bellsorp-mini) using the Brunauer-Emmett-Teller (BET) method.

3. Fabrication of cells

3.1 Fabrication of composite Li metal anodes

A facile molten infusion method at 300 °C was applied to embed molten Li into ZnO/CNF and MnZnO/CNF to obtain Li@ZnO/CNF and Li@MnZnO/CNF respectively. The whole process was operated in a glove box (H_2O< 0.1 ppm; O_2<0.1 ppm). In order to calculate the Li loading in Li@MnZnO-CNF, we compared the mass change before and after the Li infusion process. The mass of the MnZnO-CNF scaffold is calculated to be 2.05 mg/cm^2, while the Li@MnZnO-CNF is 5.76 mg/cm^2. Thus, the Li loading in Li@MnZnO-CNF is 3.71 mg cm^{-2} (14.3 mAh cm^{-2}).

According to the mass of Li@MnZnO-CNF, reversible areal capacity of 10 mAh cm^{-2} is equal to lithium utilization of 70% and specific capacity of 1736 mAh g^{-1}.

3.2 Assembly of cells

Half cells use the skeleton (MnZnO/CNF, ZnO/CNF) with a diameter of 10 mm as the working electrode and Li foil as the counter electrode. Symmetric cells were assembled by two Li foils or two composite Li metal electrodes with a diameter of 10 mm.

For the full cells, the $LiFePO_4$ (LFP) or $LiNi_{0.8}Mn_{0.1}Co_{0.1}O_2$ (NCM811) was employed as cathode while the fabricated composite Li metal electrode or Li foil was used as anode. The areal mass loading of LFP was about 3.5 mg cm^{-2} and the areal mass loading of NCM811 was ~ 5.0 mg cm^{-2}. The cathode diameter was 12 mm. Li-ion capacitors (LIC) were assembled utilizing PCNF as cathode and fabricated composite Li metal electrode or Li foil as anode.

In half cells and symmetrical cells, 1.0 M lithium bistrifluoro-methanesulfonylimide (LiTFSI) in DOL and DME (volume ratio 1:1) with 2% lithium nitrate was employed as the electrolyte. The electrolyte solution used in LFP full cell and LICs was 1.0 M $LiPF_6$ in ethylene carbonate (EC)/diethyl carbonate (DEC) (volume ratio 1:1). NCM811 full cells use 1.0 M $LiPF_6$ in ethylene carbonate (EC)/diethyl carbonate (DEC) (volume ratio 1:1) with 5.0 wt% Fluoroethylene carbonate (FEC) additive. All cells use microporous polypropylene film (Celgard, 2400) as the separator. The amount of electrolyte is fixed at ~50 μL.

4. Electrochemical measurements

To evaluate the Coulombic efficiency in half cells, a certain capacity of Li was deposited on the working electrode, and then Li was stripped until the voltage rose to 1.5 V.

In symmetrical cell tests, different and specific current densities and areal capacities of Li were set for measuring the electrochemical properties of the as-prepared composite Li metal electrodes.

The LFP cells were galvanostatically charge/discharge cycled between 2.5 and 4.0 V, while the charge/discharge voltage range of LICs was 2.0~4.0 V. The specific capacities of full cells and LICs were calculated based on the mass of $LiFePO_4$ and PCNF.

Galvanostatic charge/discharge analyses and electrochemical impedance spectroscopy (EIS) test were carried out using an electrochemical workstation (CHI 600, Shanghai Chen Hua Instrument Company) and Neware multichannel battery testing system (Neware Technology Ltd., China). The frequency range of EIS was 10 mHz~100kHz, while the perturbation amplitude was 5 mV (versus the open circuit potential).

Computational details

Density functional theory (DFT) was performed using the Cambridge Serial Total Energy Package (CASTEP) software [1]. The projector-augmented wave (PAW)[2] pseudopotentials and the generalized gradient approximation (GGA) in the form of Perdew-Burke-Ernzerhof (PBE)[3] exchange functional were adopted. The energy cutoff were set to 500 eV and a 3×3×1 k-point mesh was used in the sampling. The SCF tolerance was set to 1×10^{-6} eV and geometry convergence tolerance was set to 0.01 eV/Å. The MnO_2, ZnO and graphene slab were modelled with a vacuum width of 15 Å. After fully relaxation, the binding energy, E_b, were defined as $Eb=E_{total} - E_{Li} - E_{slab}$, where E_{total}, E_{Li}, and E_{slab} are the energy of MnO_2/ZnO/graphene slab binding with Li atom, single Li atom, and pristine MnO_2/ZnO/graphene slab, respectively.

[1] S. J. Clark, M. D. Segall, C. J. Pickard, P. J. Hasnip, M. J. Probert, K. Refson, M. C. Payne. Zeitschrift für Kristallographie-Crystalline Materials, **2005**, 220(5-6), 567-570.

[2] Blöchl, P. E. Projector Augmented-Wave Method. Phys. Rev. B, **1994**, 50 (24), 17953-17979.

[3] J. P. Perdew, K. Burke, M. Ernzerhof. Generalized Gradient Approximation Made Simple. Phys. Rev. Lett. **1996**, 77 (18), 3865-3868.
